# Exploring the Potential of Hierarchical Zeolite-Templated Carbon Materials for High-Performance Li–O_2_ Batteries: Insights from Molecular Simulations

**DOI:** 10.1021/acsami.3c11586

**Published:** 2023-11-16

**Authors:** Khizar Hayat, Daniel Bahamon, Lourdes F. Vega, Ahmed AlHajaj

**Affiliations:** Research and Innovation Center on CO_2_ and Hydrogen (RICH Center) and Chemical Engineering Department, Khalifa University, P.O. Box 127788, Abu Dhabi 127788, United Arab Emirates

**Keywords:** nonaqueous Li–O_2_ battery, zeolite-templated carbons, reactive force field, molecular dynamics, solid Li_2_O_2_

## Abstract

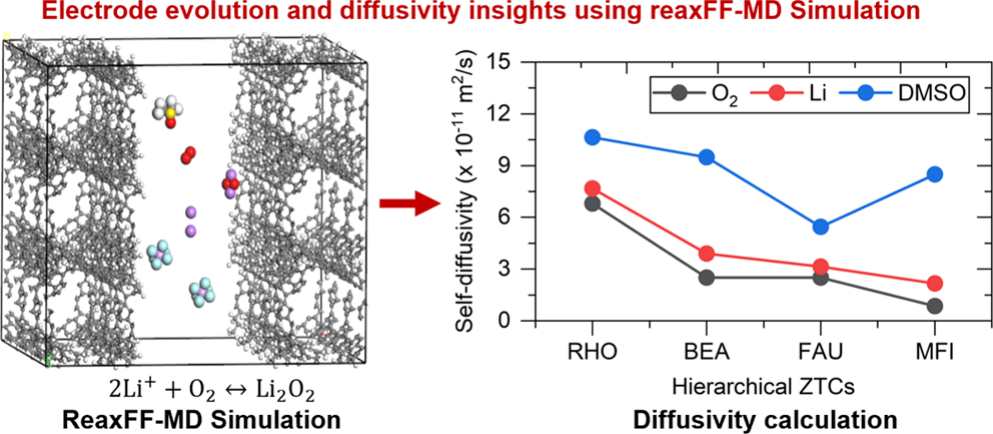

The commercialization of ultrahigh capacity lithium–oxygen (Li–O_2_) batteries is highly dependent on the cathode architecture, and a better understanding of its role in species transport and solid discharge product (i.e., Li_2_O_2_) formation is critical to improving the discharge capacity. Tailoring the pore size distribution in the cathode structure can enhance the ion mobility and increase the number of reaction sites to improve the formation of solid Li_2_O_2_. In this work, the potential of hierarchical zeolite-templated carbon (ZTC) structures as novel electrodes for Li–O_2_ batteries was investigated by using reactive force field molecular dynamics simulation (reaxFF-MD). Initially, 47 microporous zeolite-templated carbon morphologies were screened based on microporosity and specific area. Among them, four structures (i.e., RHO-, BEA-, MFI-, and FAU-ZTCs) were selected for further investigation including hierarchical features in their structures. Discharge product cluster analysis, self-diffusivities, and density number profiles of Li^+^, O_2_, and dimethyl sulfoxide (DMSO) electrolyte were obtained to find that the RHO-type ZTC exhibited enhanced mass transfer compared to conventional microporous ZTC (approximately 31% for O_2_, 44% for Li^+^, and 91% for DMSO) electrodes. This is due to the promoted formation of small-sized product clusters, creating more accessible sites for oxygen reduction reaction and mass transport. These findings indicate how hierarchical ZTC electrodes with micro- and mesopores can enhance the discharge performance of aprotic Li–O_2_ batteries, providing molecular insights into the underlying phenomena.

## 1 Introduction

In the perspective of next-generation electric vehicles, nonaqueous Li–O_2_ battery (LOB) technology (with ultrahigh energy density, i.e., >3500 Wh·kg^–1^) is touted as a potentially promising power technology and a successor of current Li-ion battery systems. The fundamental difference between Li-ion and Li–O_2_ batteries lies in how the Li^+^ ions are stored within the porous cathode: in Li-ion battery cells, Li^+^ are stored through weak intercalation interactions with the layered cathode structure resulting in a lower energy storage. On the Li–O_2_ battery, the energy is stored through the chemical bonds’ formation between Li^+^ and O_2_, which are stronger than the intercalation attractions and might store a maximum amount of energy. Further, the use of oxygen as an electrochemical reactant in the Li–O_2_ battery makes it lightweight and compact to deliver more energy density for long-range electric vehicles (EVs). The electrochemical reactions occurring inside the cathode pores during discharging and charging of nonaqueous Li–O_2_ cells, respectively,^1^ are:
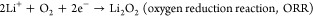
1
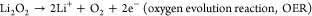
2Early studies^2−4^ reported that the oxygen reduction reaction (ORR) discharging mechanism may generate large-sized toroids of an insoluble lithium peroxide (Li_2_O_2_) discharge product, resulting in limited mass transport and a lower discharge capacity. Even though the specific deposition/dissolution mechanism of the product that limits the discharge capacity is still unclear, it is widely acknowledged that utilizing a tailored porous cathode structure combined with a high Li_2_O_2_ solubility electrolyte (i.e., DMSO) may promote the mass transport properties, leading to the maximum discharge capacity.^5,6^

To date, carbon-based materials,^7^ such as carbon nanotubes (CNTs),^8,9^ carbon nanomaterials (CNMs),^10^ and hierarchical porous carbons (HPCs),^11−15^ are widely used in energy storage systems for nonaqueous Li–O_2_ batteries due to their excellent electrical conductivity, large surface areas, and lightweight, resulting in an efficient diffusion of Li^+^ and O_2_. Despite their advantages, carbon electrodes (CNTs and CNMs) facilitate the solution-mediated formation of Li_2_O_2_ toroids, which limits their discharge capacity, making it crucial to understand the reaction mechanism as well as transport phenomena inside the porous carbon framework. In this regard, HPCs generated from the precise manipulation of porous structures have gained much attention to accommodate more Li_2_O_2_ discharge products within the hierarchy of limited void space. Numerous porosities^11,16−18^ in the micro, meso, and macro size ranges in hierarchical carbon cathodes offer shape selectivity for discharge products or intermediates and facilitate the mass transport. Because of this, it is essential to precisely tailor porous materials to produce high-performance HPCs with a distinctive micromeso-macroporous structure, which contributes to an improved discharge capacity of Li–O_2_ batteries.

Numerous experimental studies have been conducted focusing on designing novel hierarchical cathode materials for an ultrahigh discharge capacity. For instance, Li and co-workers^19^ reported a method for the fabrication of micrometer-sized honeycomb-like hierarchical carbon electrodes utilizing hard templates of nano-CaCO_3_, which delivered a much higher discharge capacity (5862 mAh·g^–1^) for the Li–O_2_ battery. Moreover, Wang et al.^20^ developed free-standing, hierarchical carbon structures from graphene oxide (GO) gel using the *in situ* sol–gel technique. The maximum discharge capacity obtained from such a structure was approximately 11,060 mAh·g^–1^. Further, Xiao and co-workers^21^ fabricated a novel electrode from the unique hierarchical arrangement of functionalized graphene sheets in the absence of electrocatalysts. This porous cathode delivered an exceptionally high recorded discharge capacity (∼15,000 mAh·g^–1^), which was attributed to facilitated Li^+^ and O_2_ transport and effective pore utilization of interconnected meso-macropores.

Apart from HPCs, the use of a novel class of ordered microporous zeolite-templated carbons (ZTCs) is widely acknowledged among various energy storage applications,^22−24^ including Li-ion, Li–S, sodium, and aluminum batteries. ZTC-based materials are synthesized by using zeolites, which are crystalline aluminosilicates with uniform pore sizes and shapes. During the synthesis process, the zeolite template is used to create a carbon replica with a pore size and shape similar to those of the zeolite template. ZTCs have highly ordered, uniform pore structures with micropore sizes, which make them suitable for energy storage purposes. However, the scope of ZTCs in Li–O_2_ batteries has not been explored yet, and it would be of paramount importance to utilize tailored ZTC structures as potential air electrodes for LOB cells owing to their high surface areas, porosities, and catalytic activity. In this regard, modeling schemes^25^ would be critical for the optimal design of hierarchical ZTC electrodes by regulating structural characteristics such as porosity, pore size and distribution, and pore connectivity in a controlled manner, which in turn could improve the performance through facilitating mass transport and discharge product distribution and enhancing structural conductivity and stability.

Although ZTC materials show promise for energy storage applications, their development has been hindered compared to other hierarchical porous carbons due to a limited number of successfully synthesized ZTCs. To address this issue, Braun et al.^26^ recently developed an efficient computational approach that accurately generates microporous ZTC structures at the atomic level, including both previously known and novel structures, opening the door to rigorously investigate the potential of ZTC structures for high-performance Li–O_2_ batteries.

The work presented here is the first study devoted to screening 47 promising ZTC structures as a cathode material for a Li–O_2_ battery. We performed a detailed screening of their structures based on microporosity and specific surface area to select the top four ZTCs for further investigation. The selected four ZTCs (RHO, FAU, MFI, and BEA) were further utilized to generate hierarchical structures and then studied using reactive force field molecular dynamics simulation (reaxFF-MD) to explore the reactive mass transport of Li^+^, O_2_, and the electrolyte solvent (DMSO) and the discharge product distribution on such ZTC electrodes. This work highlights a comprehensive computational study to rationally design hierarchical ZTC materials as potential electrodes for next-generation Li–O_2_ batteries, aiming at improving their performance.

## 2 Methodology

### 2.1 Design and Characterization of ZTC-Type Cathode Structures

ZTC structures are synthesized using the templating technique in which pyrolytic carbonization of either ethylene, ethene, or propylene inside the zeolite template pores results in three-dimensional (3D) microporous ZTCs.^27−32^ Computationally, molecular simulations including grand-canonical Monte Carlo (GCMC)^26^ have been employed to generate ZTC from the zeolite template by mimicking the experimental methods.^33,34^ In the GCMC algorithm, the sp^2^-hybridized carbon atoms are inserted following the Monte Carlo (MC) moves, which permits the newly added carbon atoms to find their optimal position inside the zeolite template. The MC moves are either accepted or rejected based on the energy minimization criterion. The carbon loading process stops once there are no more surface binding sites available. The main advantage of this computational scheme is that neither it relies on experimental data nor is it computationally expensive. Hence, it can be extended to a variety of zeolite templates to model several novel ZTCs.^26^

Here, we adopted the 47 ZTC structures (Table S1) from Braun et al.’s^26^ work and generated them using the GCMC scheme. The first screening was performed based on the geometric characterization of the structures. Pore Blazer 4.0, an open-source package available online from the github repository (https://github.com/SarkisovGroup/PoreBlazer), was used for this purpose. During the structural analysis, nitrogen was employed as the probe molecule to produce the data such as pore volume, accessible surface area, large-cavity diameter (LCD), and pore-limiting diameter (PLD) in the ordered microporous zeolite-templated carbon structures. The involved algorithms for computing various geometric informatics are discussed elsewhere in detail.^35^

**Figure 1:**
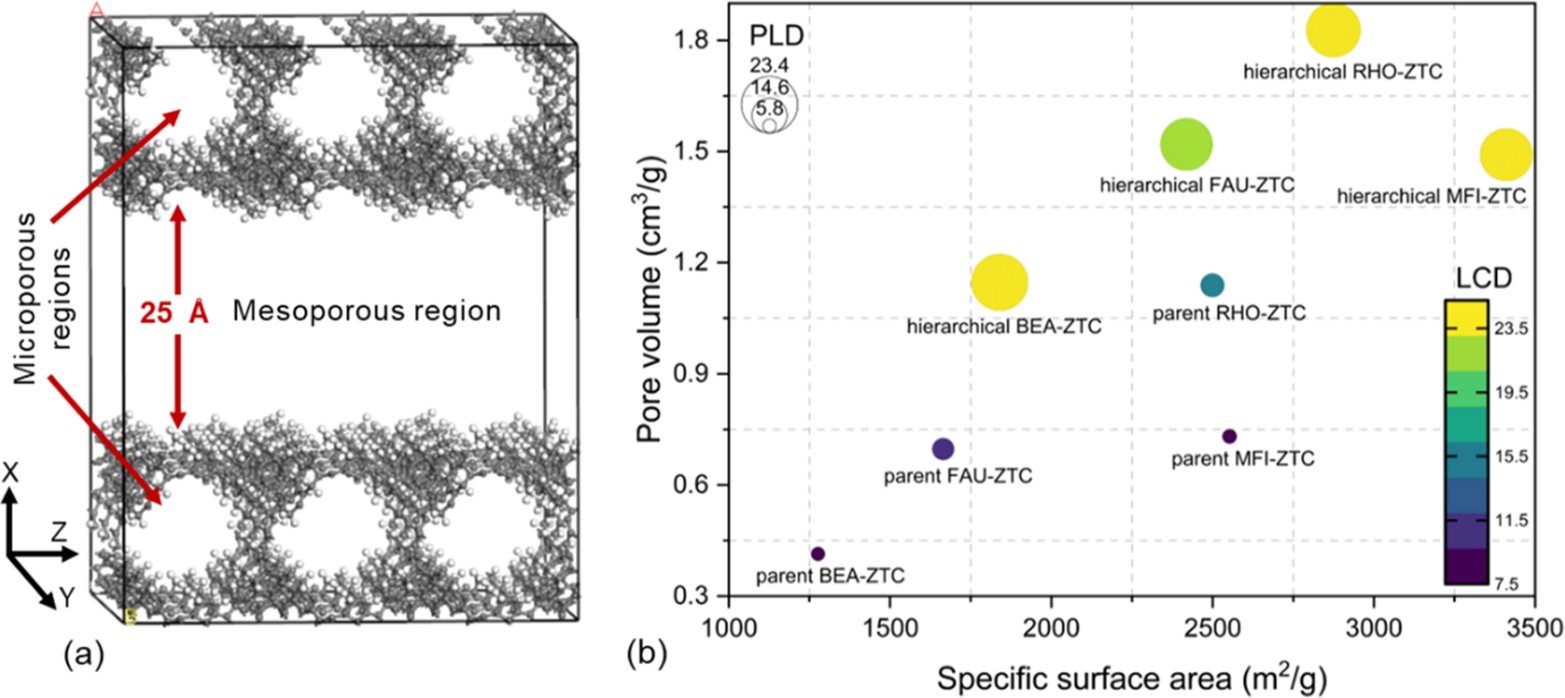
(a) Schematic illustration of hierarchical ZTCs containing micro- and mesoporous regions [color code: gray and white for carbon and hydrogen atoms, respectively] and (b) 2D plot of structural properties of selected hierarchical ZTCs and their intrinsic microporous structures.

Considering mass transport accessibility limitations and various properties spotted on a two-dimensional (2D) plot (see Figure S1), we have selected four ZTC structures for further investigation, including RHO-ZTC, MFI-ZTC, FAU-ZTC, and BEA-ZTC (obtained from RHO, MFI, FAU, and BEA-type zeolites, respectively) to cover the range of structural properties. Initially, supercells of the considered ZTC structures were generated from their respective intrinsic microporous unit cells. Next, to create hierarchical frameworks of the constructed supercells (i.e., containing both mesoporous and microporous regions), a slit-shape mesopore (of size 25 Å, along the *x*-direction) was introduced into the center of the ordered microporous supercells by carving part of the replicated structure.^25^ Carbon terminations were filled with H atoms. The resulting hierarchical ZTC structure is composed of a single mesoporous region sandwiched between two microporous regions, as illustrated in Figure 1a. The scheme of developing simulation boxes for all of the hierarchical ZTCs is presented in Figure S2. All of the four ZTCs comprise the same-sized (25 Å) mesoporous region; however, microporous regions with varying thicknesses such as 17.3, 18.0, 14.0, and 12.8 Å for RHO-, MFI-, FAU-, and BEA-ZTCs, respectively, were obtained. Eventually, the different thickness values are dependent on the respective structural dimensions, which are different for all of the ZTC structures (see Figure S2). Despite the fact that different thicknesses of microporous regions may impact structural performances, we still performed comparative analysis of such structures to just obtain a general understanding about their performance as Li–O_2_ battery electrodes. Further, we computed the structural properties such as pore volume, surface area, LCD, and PLD for the hierarchical structures. As demonstrated by the 2D plot shown in Figure 1b, unlike microporous ZTCs, the inclusion of a mesoporous region significantly improves all of the corresponding structural characteristics.

For the characterization of hierarchical ZTCs (i.e., pore size distribution), we calculated N_2_ adsorption isotherms at 77 K using GCMC simulations (using RASPA^36^) by keeping constant the chemical potential, volume, and temperature of the simulation box, while the number of N_2_ molecules varied depending on the pressure. The N_2_ adsorption isotherm was obtained by taking an average of the number of N_2_ molecules adsorbed at various pressure ratios. To determine the Lennard-Jones potentials between the adsorbed nitrogen molecules and carbon atoms of the ZTC structure, the interaction parameters are taken from the transferable potential for phase equilibria (TraPPE) force field.^37^ The cutoff radius was set at 13 Å, and the charges were computed using the Ewald summation method with a precision level of 1 × 10^–6^. The whole GCMC simulation spanned three stages, including the initialization (∼1000 cycles), equilibration (∼4000 cycles), and production (∼10,000 cycles) steps, which were demonstrated to be enough to obtain the equilibrated adsorption isotherms.

### 2.2 ReaxFF-MD Simulations of Hierarchical ZTC Electrodes

**Figure 2:**
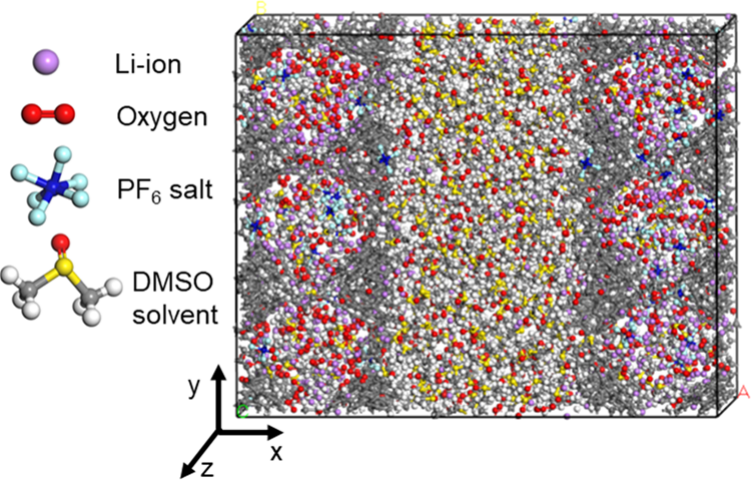
ReaxFF-MD simulation box based on hierarchical RHO-ZTC.

In order to obtain physical insight into the transport properties and discharge product growth (Li_2_O_2_) within the hierarchical ZTC (and their parent versions) cathodes of the Li–O_2_ battery, we performed reactive force field molecular dynamics simulation (reaxFF-MD) by means of the LAMMPS^38^ package. The initial configurations of lithium, oxygen, and electrolyte for the Li–O_2_ battery electrodes were generated using the amorphous cell module from BIOVIA Materials Studio software. First, we filled the hierarchical ZTC cathodes by randomly placing electrolyte (i.e., DMSO) molecules, as shown in Figure 2. The number of electrolyte molecules in each structure was adjusted to maintain the bulk electrolyte density (i.e., 1.09 g·cm^–3^).^39^ In addition, Li^+^ and PF_6_^–^ ions were included to resemble a LiPF_6_ solution of 1 M concentration. Further, various numbers of gaseous O_2_ (100, 200, 300, 400, and 500 molecules) were dissolved in the electrolyte solution to obtain different temporal configurations of hierarchical ZTCs. Here, the different number of oxygen molecules mimics the discharge process stages, with 100 being the initial stage and 500 being the final stage of the discharge process. Notably, it is assumed that all of the dissolved oxygen is completely reduced to reactive oxygen peroxide species, i.e., O_2_^2–^. A comprehensive detail of the number of initial species in all of the hierarchical ZTC electrodes is listed in Table 1. As shown in the table, the number of Li ions used is equal to the sum of the counteranions (PF_6_^–^ and O_2_^2–^) to neutralize the system.

**Table 1:** Hierarchical ZTCs Studied in the ReaxFF-MD Simulations^a^

species	RHO					FAU				
DMSO	567	567	567	567	567	407	407	407	407	407
PF_6_	41	41	41	41	41	30	30	30	30	30
Li	241	441	641	841	1041	230	430	630	830	1030
O_2_	100	200	300	400	500	100	200	300	400	500
system density (g·cm^–3^)	1.111	1.168	1.225	1.282	1.338	1.122	1.192	1.263	1.333	1.404

species	MFI					BEA				
DMSO	386	386	386	386	386	461	461	461	461	461
PF_6_	28	28	28	28	28	34	34	34	34	34
Li	228	428	628	828	1028	234	434	634	834	1034
O_2_	100	200	300	400	500	100	200	300	400	500
system density (g·cm^–3^)	0.989	1.046	1.103	1.160	1.217	1.189	1.247	1.305	1.363	1.421

a Number of molecules per super cell.

To compute the molecular interactions, the empirical reaxFF was utilized with the parameter set for the elements C, H, O, Li, P, F, and S.^40^ Besides, to compute discharge product formation (Li_2_O_2_), the parameters for Li and O were replaced with the one developed for lithium–oxygen systems.^41^ Unlike the typical classical force field, the reaxFF is a bond order (BO)-based method which allows bond breakage and generation during MD simulations.^42−44^ In reaxFF-MD, the total system energy can be calculated mathematically according to eq 3, as follows:
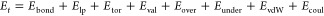
3where, *E*_t_, *E*_bond_, *E*_lp_, *E*_tor_, *E*_val_, *E*_over_, *E*_under_, *E*_vdW_, and *E*_coul_ are total system, bond, lone pair, torsional, valence, overcoordination, undercoordination, van der Waals, and Coulombic energies, respectively. During reaxFF-MD simulations, bond order values are computed at every time step from interatomic bond distances, consequently acting as a prime component for all types of bonded interactions, such as torsional and valence interactions. Once the bond cleavage happens, all of the bonded energy contributions are diminished, leaving behind only long-range nonbonded interactions including van der Waals and Coulombic terms, normally present among the atomic pairs that are not bonded. Though the reaxFF has been successfully employed in several studies to simulate reactive environments,^45^ the charge equilibration is still a major bottleneck. The electronegativity equalization method (EEM), which is a geometry-dependent scheme, is used to equilibrate the molecular charges. This mainly leads to unreasonable charge distribution for the molecules restricting the proper utilization of reactive force fields. To prevent this issue, we have used constant charges in this work for all of the constituent molecules. The atomic charges for DMSO and PF_6_^–^ are taken from the COMPASS force field.^46^ In the atomic charge assignment, Li is set at 1 (i.e., Li^+^) and O_2_ is set at −2 (i.e., O_2_^2–^). This choice is underpinned by two primary considerations: first, to replicate the influx of electrons into the cathode during discharging, and second, to facilitate the production of the Li_2_O_2_ discharge product, as evidenced in experimental studies. The precise charge assignment, particularly about oxygen molecules, is of paramount importance as it can significantly influence the generation of discharge products contingent upon the availability of electrons (equivalent to the O_2_ charge). Nevertheless, further exploration is needed to fully comprehend the impact of applied charges on the types of products generated.

The simulation cells were initially optimized by using the conjugate gradient scheme. The value for energy and force convergence criteria was set at 1 × 10^–10^ to eliminate unwanted bond formation and atomic overlapping in the simulation box. Then, reaxFF-MD simulations were performed in the NVT ensemble with a Nosé–Hoover thermostat for temperature control. A value of 0.1 fs was used for the time step. In the first steps, we performed a short MD equilibration at 15 K for 10 ps to remove any existing hot spots in the optimized geometries. Then, we heated the systems from 15 to 300 K (target temperature) upon 10 ps with a heating rate of 28.5 K·ps^–1^. This was followed by further equilibration at 300 K. Then, a production stage was run for 1.5 ns for all systems. During the production run, we dumped trajectories at every 100 fs to postprocess and analyze the discharge product (Li_2_O_2_) evolution with time. Periodic boundary conditions were applied in all three directions. Ovito software^47^ was used for postprocessing and visualization of the output simulation-generated trajectories. Different properties, such as density profiles, cluster analysis, and self-diffusion coefficients, were obtained from the MD simulations.

For diffusion analysis of species, we have computed the mean-squared displacement (MSD) based on the position of diffusing molecules (see eqs 4 and 5) and used the Einstein’s relation^43,48^ to obtain the self-diffusion coefficients through the preferential direction.
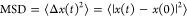
4
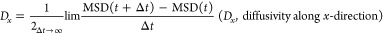
5

The self-diffusion coefficients, for the case of Li^+^ and O_2_, correspond to the total number of Li^+^ and O_2_, respectively, present either in intermediates (such as LiO, Li_2_O^+^, and LiO_2_^–^) or in discharge product (Li_2_O_2_) clusters.

The cluster analysis was based on an analysis of the dumped trajectories. Particles were distinguished into various clusters as a function of selected neighbors’ criteria, including topology criteria (bond length-based) and distance criteria (cutoff range-based). Here, we have considered the former cluster analysis criteria (i.e., topology) for which a bond distance value of 2.56 Å^49^ was defined to form a cluster between Li^+^ and O_2_^2–^. The clusters containing only single atoms, two atoms, or straight chains were excluded from the analysis.

## 3 Results and Discussion

### 3.1 Hierarchical ZTC Structural Characterization and Stability

**Figure 3:**
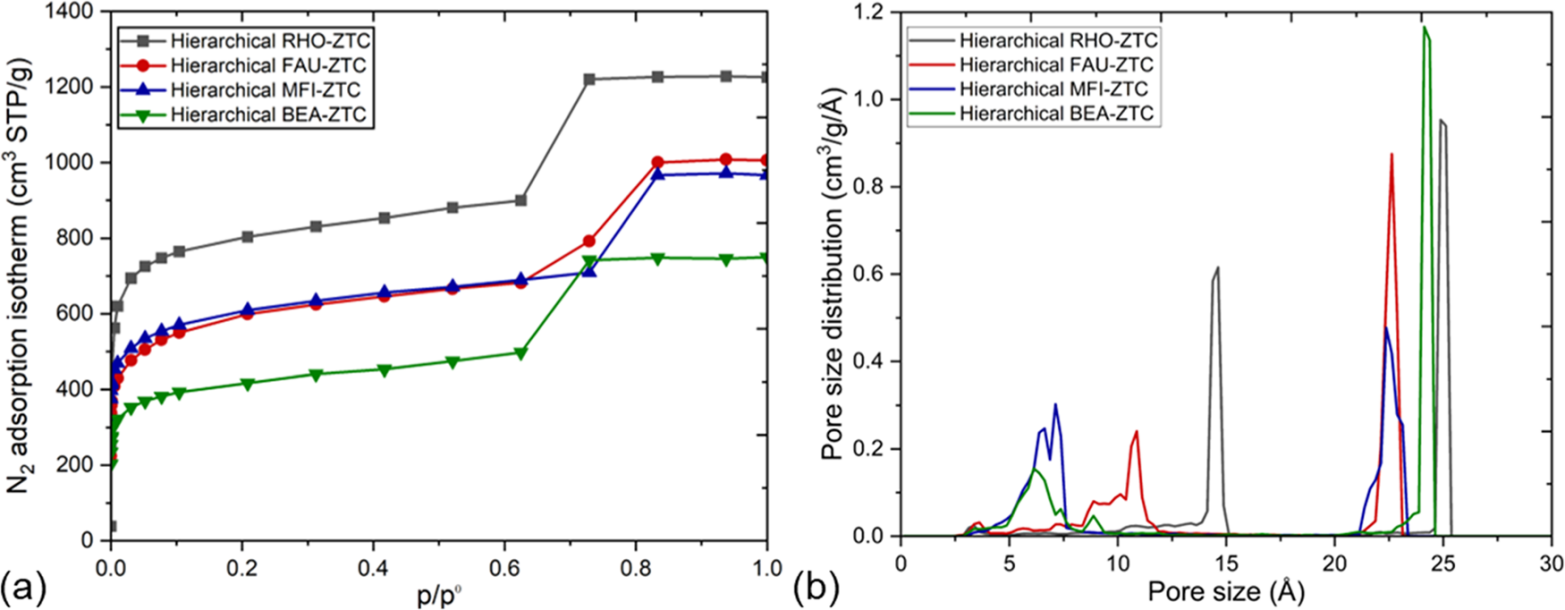
Description of (a) N_2_ adsorption isotherms of developed hierarchical ZTCs (error bars smaller than the symbols) and (b) resulted pore size distributions.

Figure 3a demonstrates the nitrogen adsorption isotherms and pore size distributions of the hierarchical RHO-ZTC, FAU-ZTC, MFI-ZTC, and BEA-ZTC structures. These isotherms were calculated within the relative pressure (*p*/*p*^0^) range of 1 × 10^–5^ – 1.0 using GCMC. Type IV isotherms were obtained in all cases.^50^ Among the hierarchical ZTCs, the RHO-type ZTC exhibited maximum amount of adsorbed nitrogen in both microporous and mesoporous regions, which is due to the presence of large-sized interconnected cavities (∼14.5 Å) in the microporous region. Moreover, for all of the structures, at low relative pressures, the adsorption occurs mainly in the microporous region, which initially results in a steep rise in nitrogen adsorption and then steadily increases until the relative pressure value reaches c.a. 0.6. As the relative pressure increases further, the slit-type mesoporous region starts to fill up, leading to a jump in the isotherm shape. The rise in adsorption at intermediate relative pressures (i.e., 1 × 10^–5^ – 0.6) can be explained by the capillary condensation phenomenon, which occurs when the pressure is high enough for the nitrogen adsorbate to fill the micropores. At this point, the adsorbate molecules start to condense and form a liquidlike phase in the pores, leading to the rise in the adsorption isotherm. The plateau region (i.e., 0.6–1.0) in the isotherm is due to the filling of the mesopore, which is typically larger (∼24.5 Å) than the micropores. The adsorbate molecules in the slit-type mesopore form multilayers on the surface, resulting in a plateau in the adsorption isotherm. The thickness of the multilayer depends on the size of the slit-type mesopores and the temperature.

The pore size distribution results of the adsorption isotherms are plotted in Figure 3b. All of the hierarchical ZTCs indicated two peaks corresponding to the microporous and mesoporous regions. The mesoporous peak for all structures is located around 24.3 Å, whereas the microporous peaks for RHO-, FAU-, MFI-, and BEA-ZTCs are 14.5, 10.6, 6.8, and 6.6 Å, respectively, due to the presence of varying sized interconnected cages and channels inside the microporous cathode walls. Moreover, it is evident that the minimum pore size in any hierarchical ZTC structure is 6.6 Å, which is large enough to allow the penetration of a bulky molecule of LiPF_6_ (∼5.10 Å) into the microporous structures.

**Figure 4:**
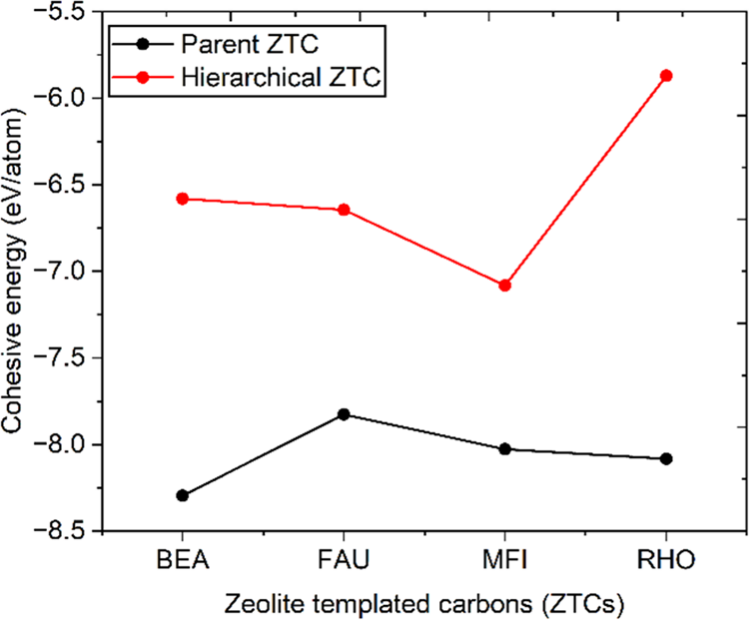
Cohesive energy trends of the studied hierarchical ZTCs and their parent microporous structures.

Furthermore, the structural stability of the generated hierarchical ZTCs is assessed by analyzing the cohesive energy. Cohesive energy is defined as the energy required to disassemble a structure into neutral free atoms at an infinite separation. It can be represented mathematically as^51^
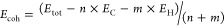
6where, *E*_coh_, *E*_tot_, *E*_C_, and *E*_H_ are the cohesive, total system, individual carbon (in the diamond crystal), and hydrogen atom (in the hydrogen molecule) energies, respectively. *n* and *m* are the number of carbon and hydrogen atoms in the structures, respectively. A negative cohesive energy value signifies structural stability of the structure, with larger negative values indicating greater stability, while smaller negative values imply less stability. As illustrated in Figure 4 and Table S2, the calculated cohesive energies of the developed hierarchical ZTCs are negative, supporting the feasibility of developing such hierarchical designs. It has been observed that hierarchical architectures exhibit smaller negative values compared to their parent microporous structures, indicating that hierarchical structures are comparatively less stable. Nevertheless, the negative cohesive energies of hierarchical designs confirm their potential for their construction. Remarkably, the hierarchical ZTCs have shown satisfactory cohesive energies compared to previously developed Zeo-C and graphene-based structures.^52^

This leads us to assert with confidence the promising feasibility of experimental synthesis for our hierarchical designs, which could yield outstanding results in energy storage applications. Recent advancements have been made in the development of hierarchical ZTCs by utilizing zeolites as sacrificial templates.^53^ Leveraging the mesoporosity introduced by the surfactant-templated zeolite has enabled the creation of a hierarchical ZTC with a significant mesopore volume (i.e., 0.85 cm^3^ g^–1^), comparable to our proposed hierarchical structures (i.e., 0.69–0.88 cm^3^ g^–1^). Additionally, the rearrangement of surfactant-templated mesoporosity has been shown to influence pore size distributions and textural characteristics, which can be confirmed through nitrogen adsorption isotherms and X-ray diffraction analysis. Encouragingly, this approach holds promise for the experimental synthesis of various hierarchical ZTC designs.

### 3.2 Diffusion Coefficients through Hierarchical ZTCs

According to experimental and macro-modeling investigations, LOBs with a hierarchical electrode with different pore sizes such as nano-micro, micro-meso, and meso-macro have higher discharge capacities than their equivalent counterparts with single pore size.^21,25,54−62^ The mass transfer of oxygen through the hierarchical structure, where large pores serve as carriage ways for oxygen transport and small pores are used to distribute and store the Li_2_O_2_ discharge product, is thought to be the primary factor in this improved performance. Using RHO-, FAU-, MFI-, and BEA-ZTC, we performed reaxFF-MD simulations to evaluate the reactive transport of the Li–O_2_ battery species at the molecular level and provide insights into the respective discharge mechanism.

**Figure 5:**
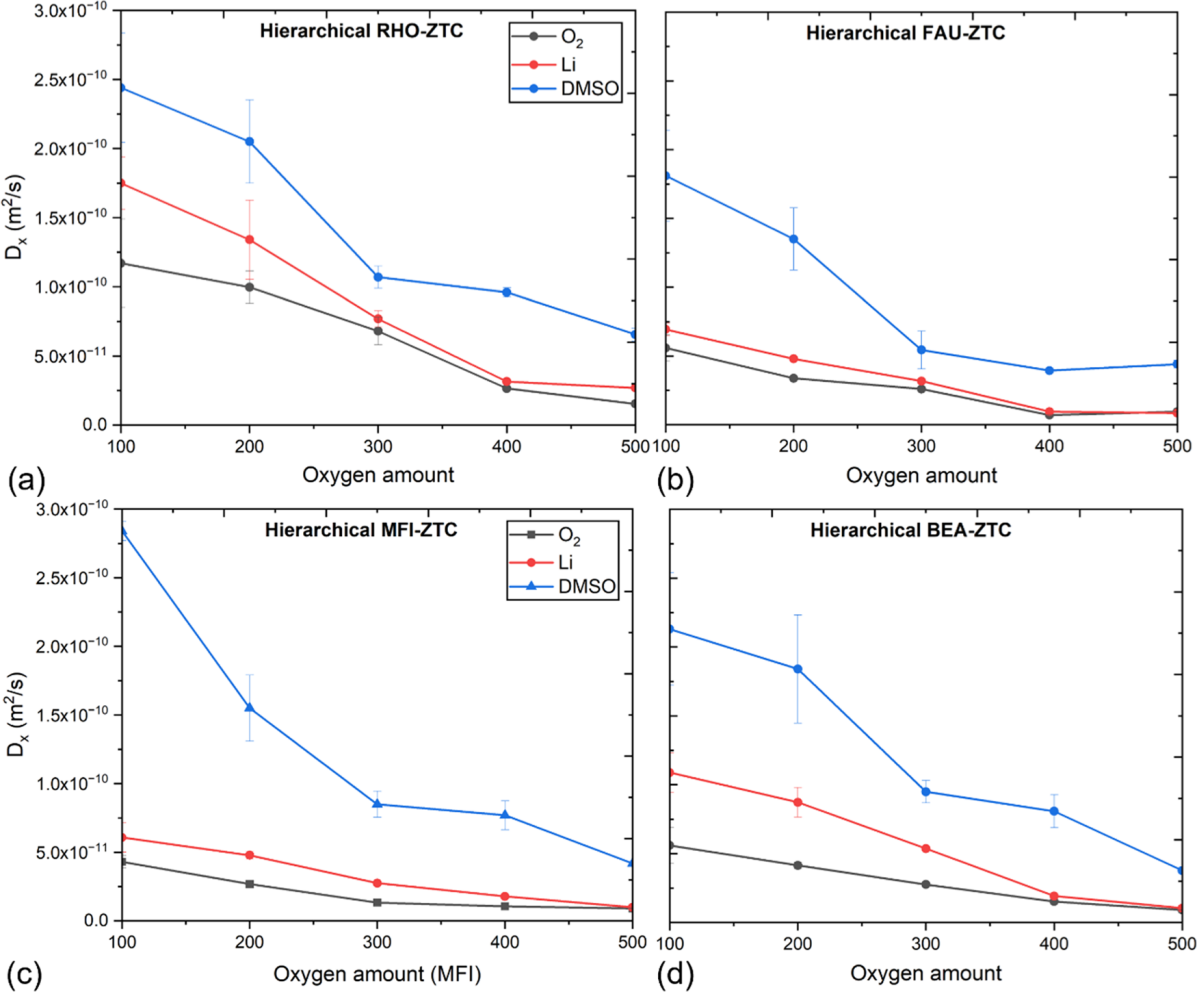
Self-diffusivity (*x*-direction) trends for oxygen, lithium ions, and DMSO electrolyte at various discharge states through hierarchical (a) RHO-ZTC, (b) FAU-ZTC, (c) MFI-ZTC, and (d) BEA-ZTC.

Figure 5a–d and Table S3 in the Supporting Information illustrate the influence of discharge product (Li_2_O_2_) growth on the *x*-direction (the preferred diffusion route toward reaction centers in microporous walls) self-diffusivities of oxygen, Li^+^, and DMSO inside RHO-, FAU-, MFI-, and BEA-ZTC electrodes. It was observed that all species’ self-diffusivities decreased significantly as the discharge process progressed, i.e., as the number of reactive oxygen (O_2_^2–^) molecules increased from 100 to 500. This is due to the continuous growth of the discharge product (Li_2_O_2_) aggregates, which not only reduce the quantity of free lithium ions and oxygen molecules but also increase their transport resistance. At the beginning of discharging (i.e., 100 reactive oxygen molecules), the diffusivities of Li^+^ and reactive oxygen are large, indicating the existence of small reactive intermediates (e.g., LiO, Li_2_O^+^, LiO_2_^–^) and small discharge product (Li_2_O_2_) clusters inside the hierarchical ZTC electrode framework. At the end of the discharge process (i.e., 500 reactive oxygen molecules), large discharge product clusters are formed, which are strongly adsorbed to the electrode surface and fill the entire pore volume resulting in reduced self-diffusivities of Li^+^ and oxygen.

In all hierarchical ZTCs, the lithium-ion diffusivity is greater than the oxygen diffusivity, indicating that the diffusivity of Li ions is governed not only by reactive oxygen but also by salt ions (i.e., PF_6_^–^). Initially, PF_6_^–^ salt ions influence the movement of Li ions, causing a difference in diffusivity between lithium and oxygen within 100–300 reactive oxygen consumption. However, at 400–500 oxygen molecule consumption, this difference diminishes because nearly all Li ions and oxygen have completely reacted and formed clusters at this stage. Among all structures, hierarchical RHO-ZTC revealed a superior performance, most likely as a result of the huge pore volume (1.85 cm^3^·g^–1^) and high density of micro- (14.5 Å) and mesopores (25 Å), which not only store a significant amount of solid product, but also gives enough room for species transfer without pore blockage. In addition, the high density of micro- and mesopores inside hierarchical RHO-ZTC have promoted the interdiffusion of species between mesoporous and microporous regions. This finding supports the argument stated by Elabyouki et al.^25^ that the better discharge characteristics of hierarchical electrodes are due to the constant transfer of species between the mesopore and micropores, which prevents the micropores from clogging during discharging. Even though other hierarchical ZTCs (FAU, MFI, and BEA) have also observed interpore transfer, it is negligible in comparison with hierarchical RHO-ZTC.

For the case of the electrolyte solvent (i.e., DMSO), the self-diffusivity decreased in a more notorious way than Li^+^ and O_2_^2–^ as the discharge mechanism proceeded. This is due to the discharge product formation, which limits the transport of bulkier DMSO molecules (diameter size: ∼5 Å) through the blocked small cages and interconnected channels of the hierarchical ZTCs. Therefore, at large oxygen consumption, electrolyte solvent movement is mostly restricted to the mesopore, particularly in hierarchical MFI-ZTC. In addition to the *x*-direction self-diffusivity, we have also reported the overall diffusivities (average of *x*, *y*, and *z* directions) of Li^+^, O_2_, and DMSO, which are tabulated in Figure S3 and Table S4. It is observed that hierarchical RHO-ZTC shows noticeably less reduction in both overall and *x*-direction diffusivities through the course of the discharging process. For instance, the obtained overall diffusivity ranges (at O_2_ = 100–500) in hierarchical RHO-ZTC for O_2_ and Li^+^ are 1.17 × 10^–10^–1.59 × 10^–11^ and 1.69 × 10^–10^–2.92 × 10^–11^ m^2^·s^–1^, respectively.

**Figure 6:**
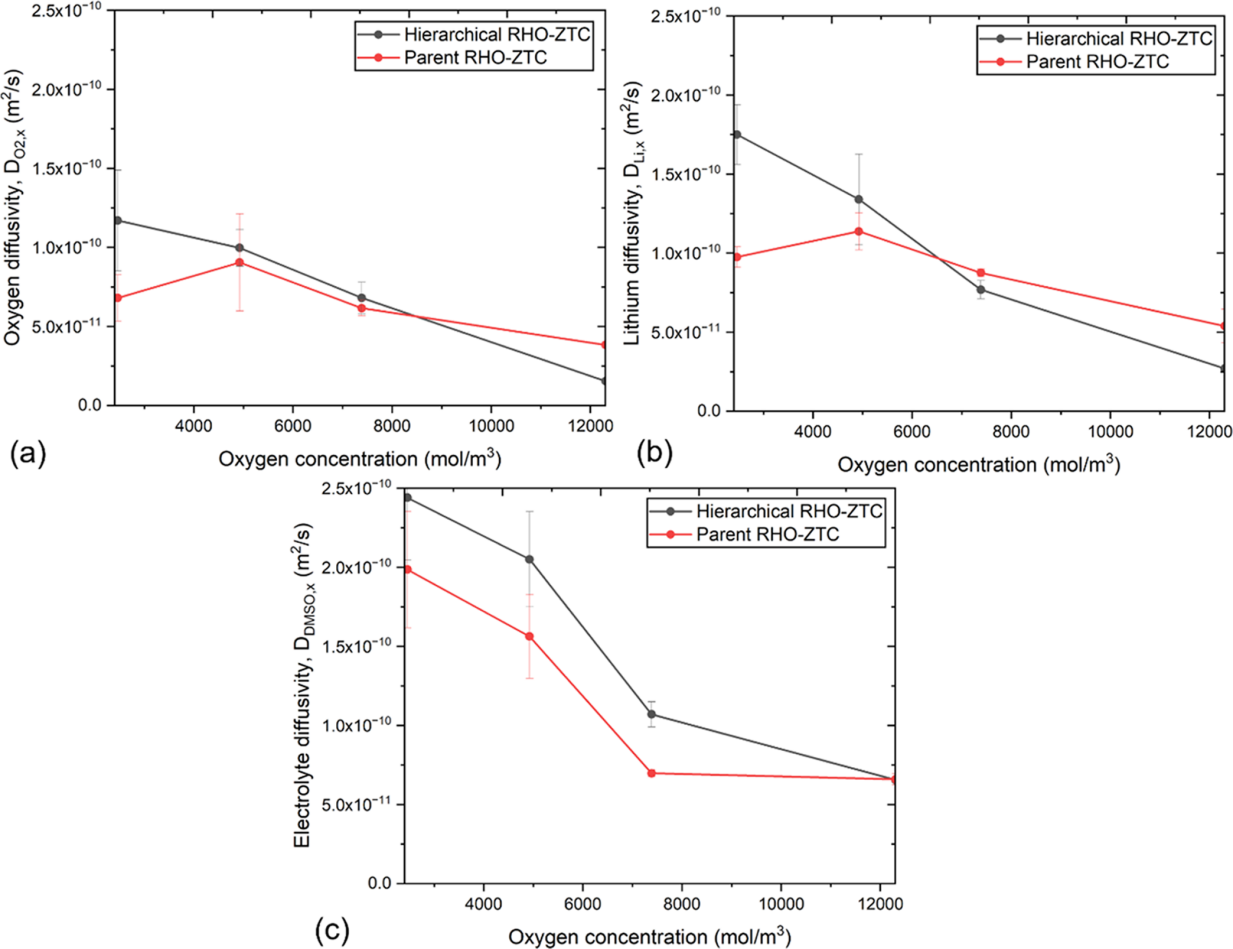
Comparative analysis of self-diffusivities of (a) O_2_, (b) Li^+^, and (c) DMSO through hierarchical and parent RHO-ZTCs.

In addition, we compared species diffusion across hierarchical and microporous parent RHO-ZTCs (as displayed in Figure 6a–c and Table S5). At a lower oxygen concentration, microporous parent RHO-ZTC exhibited approximately 0.5–1.6 times less diffusivity than hierarchical RHO-ZTC. In contrast, increased oxygen concentrations caused oxygen and lithium to diffuse more rapidly in parent RHO-ZTC than in the hierarchical one. The fundamental cause is that the parent microporous structure has less amount of electrolyte, which creates some dry spaces inside pores resulting in a faster diffusion compared to that in the hierarchical structure, which is fully loaded with liquid electrolyte. In hierarchical RHO-ZTC, however, higher oxygen concentrations promote the generation of large clusters within the microporous walls, which continue to grow within the mesopores, thereby restricting the transport of oxygen and lithium ions.

Overall, compared to the other investigated structures, hierarchical RHO-ZTC provides a superior mass transport. Previous attempts in the literature to calculate the transport of species across carbon electrodes, in particular Li^+^, have exclusively focused on nonreactive-based systems.^25^ In order to examine the bulk diffusivity of lithium ions through graphitic carbons, for example, Persson et al.^63^ developed an experimental method coupled with *ab initio* simulations. It was discovered that lithium-ion diffusion in graphite is 4.4 × 10^–10^ m^2^·s^–1^ parallel to graphene planes and 8.7 × 10^–16^ m^2^·s^–1^ perpendicular to those planes. Although their reported diffusivity is approximately 3.7 times greater than our calculated diffusivity of lithium ions using hierarchical RHO-ZTC (1.17 × 10^–10^ m^2^·s^–1^), their diffusivity was calculated without considering the counterions or reactions’ impact. Recently, our group examined the Li^+^, PF_6_^–^, and DMSO electrolyte transport within a hierarchical graphene oxide carbon electrode by using classical molecular dynamic simulations. The calculated Li^+^ self-diffusivity in the hierarchical graphene oxide carbon ranged from 3.06 × 10^–11^ to 5.5 × 10^–13^ m^2^·s^–1^. Despite ignoring the O_2_ content and reaction kinetics, the diffusivity of lithium ions was still lower than that in the hierarchical ZTCs studied in this work. This is mostly due to the graphene oxide structure’s high micropore density. The preceding explanation demonstrates that diffusion via hierarchical carbon structures can be regulated by modifying the structure’s characteristics. In this light, it is clear that ad-hoc designed hierarchical ZTC electrodes could facilitate species movement during LOB cell discharge and charge cycles.

### 3.3 Li^+^, O_2_, and DMSO Distribution

**Figure 7:**
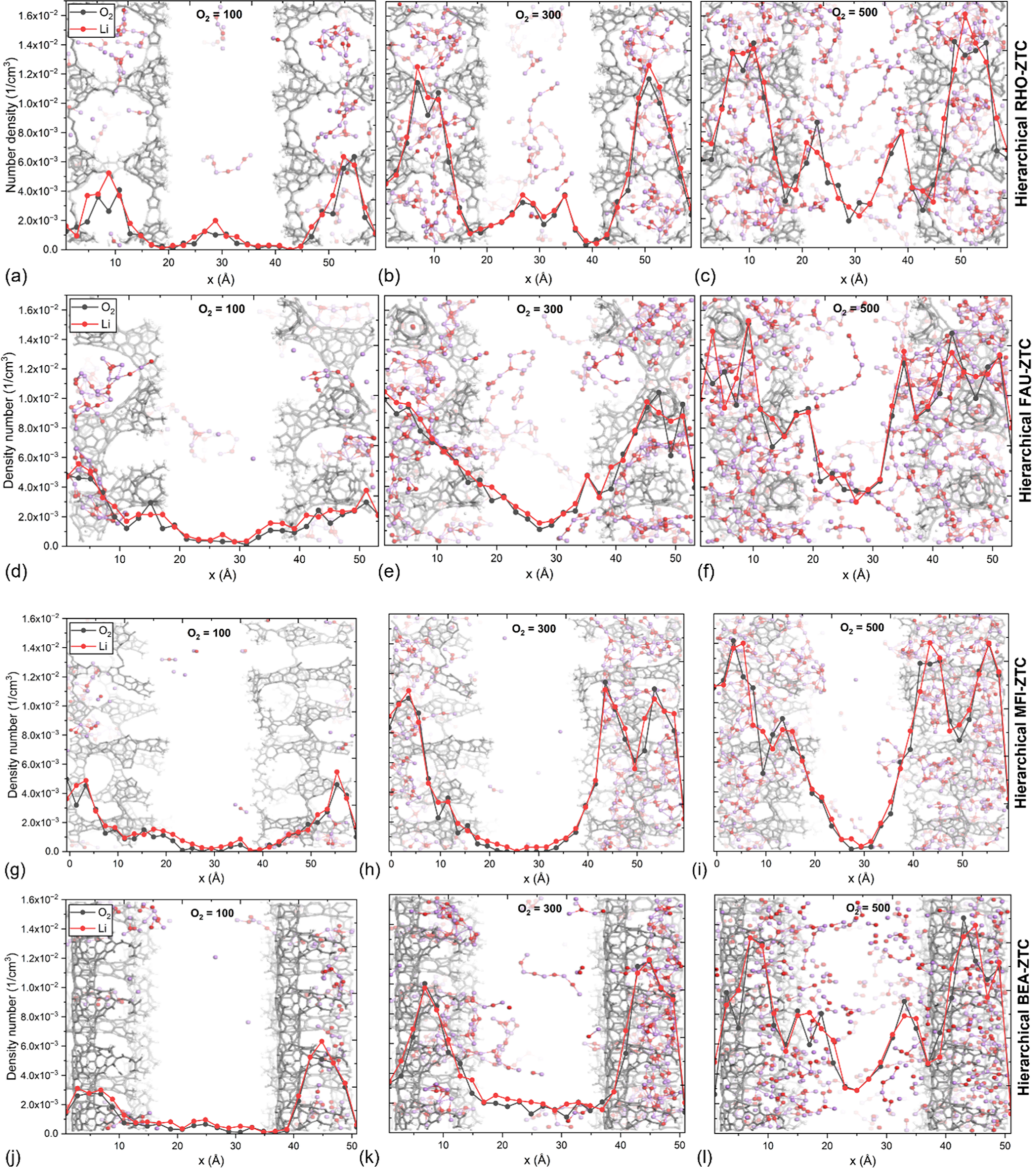
Density profiles of Li ions and oxygen along the *x*-direction at different oxygen consumptions (100, 300, and 500) through hierarchical (a–c) RHO-ZTC, (d–f) FAU-ZTC, (g–i) MFI-ZTC, and (j–l) BEA-ZTC. Snapshots after production runs (1.5 ns). Other molecules (electrolyte and PF_6_) are omitted.

We have calculated density profiles (based on the molecular center of mass along the *x*-direction) to gain insight into how hierarchical ZTC electrodes influence solid discharge product growth, its distribution, and the preferred deposition region. All hierarchical cathodes (as depicted in Figure 7) consist of a slit-type mesoporous region sandwiched between two microporous regions (left and right sides). The density profiles of specifically lithium ions and oxygen describe the distribution of the solid discharge product. Figure 7a,d,g,j exhibit the schematics of lithium ions and oxygen density profiles, respectively, across the four various hierarchical ZTCs during the initial discharge phase (i.e., O_2_ = 100). In contrast, Figure 7b,e,h,k and Figure 7c,f,i,l depict the density profiles of lithium ions and oxygen during the middle (i.e., O_2_ = 300) and final (i.e., O_2_ = 500) stages of the discharge process. The density profile plots from reaxFF-MD simulations revealed that the optimal location for electrochemical reactions and the deposition of discharge product clusters (illustrated in the background) occurs in the intrinsic microporous regions of the hierarchical electrodes, while the slit-mesoporous region serves as an oxygen transport tunnel. At the beginning of the discharge process (O_2_ = 100), the mesoporous region contains few discharge products and some intermediates (specifically in the RHO-ZTC and FAU-ZTC structures). However, as the discharge process advances, the growth of discharge product clusters inside the microporous regions also increases, leading to the growth of clusters in the mesopore, as shown in Figure 7b,e,k, and with prominent growth at the end of the discharge process (Figure 7c,f,l). Smaller pores act as reaction centers and Li_2_O_2_ deposition, whereas larger pores transport oxygen to the reaction centers during oxygen reduction reactions (ORR).^21,55,56^ These findings are consistent with the findings of multiple experimental studies claiming that smaller pores serve as reaction centers and facilitate Li_2_O_2_ deposition.

In contrast, hierarchical MFI-ZTC (Figure 7h,i) has revealed no existence of a discharge product in the mesoporous region. The underlying reason could lie on the large density of small-sized micropores (6.3–7.1 Å), which hold the discharge product clusters tightly inside the micropores and do not permit them to move out of the microporous region.

**Figure 8:**
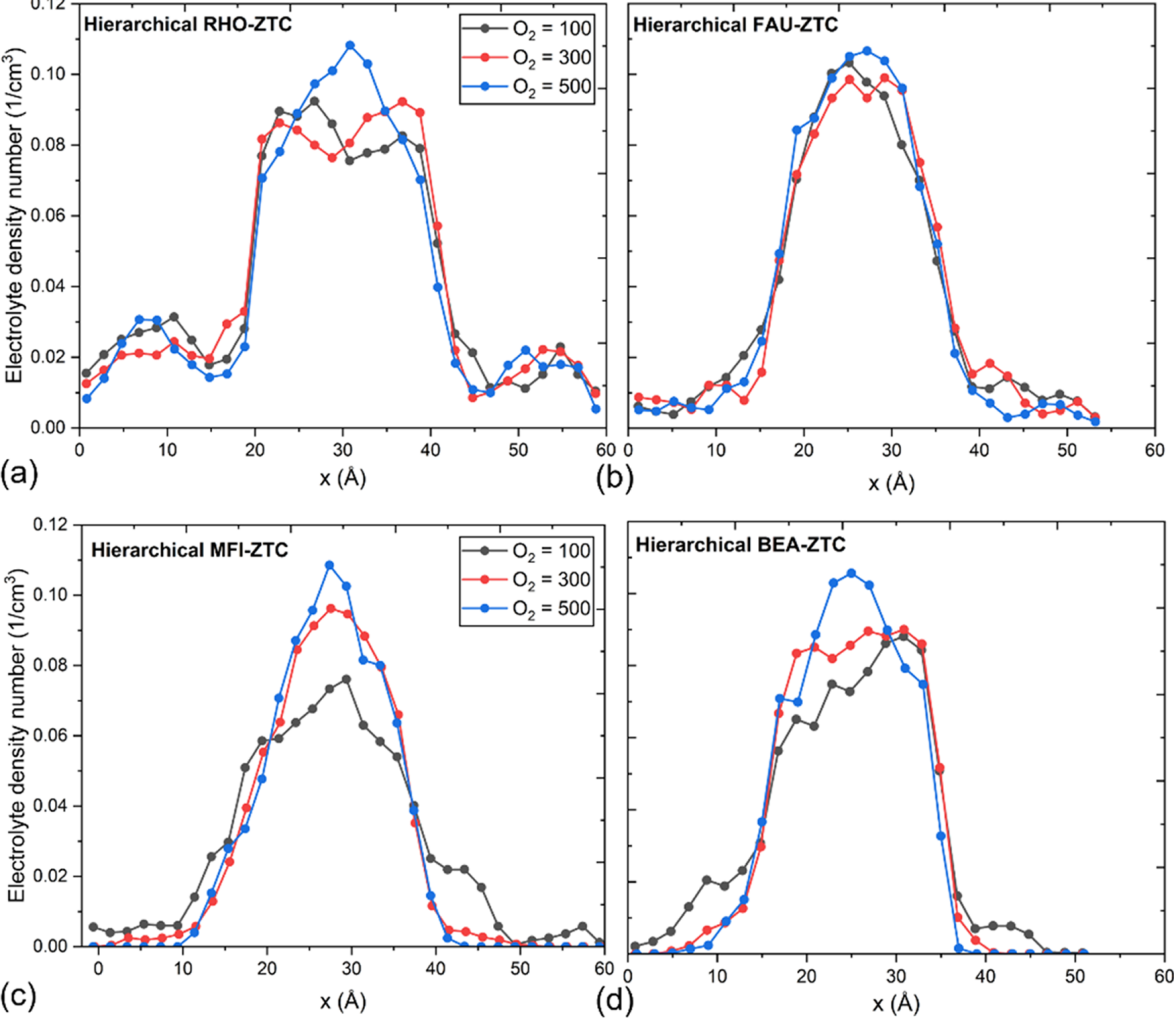
Effect of discharge product growth on the distribution of DMSO along the *x*-direction through hierarchical (a) RHO-ZTC, (b) FAU-ZTC, (c) MFI-ZTC, and (d) BEA-ZTC.

To demonstrate how the discharge product growth affects the electrolyte displacement, we plotted density number distribution curves for the electrolyte solvent, as shown in Figure 8. The amount of DMSO molecules present in the micropores is smaller compared to that in the slit mesopores because of their bulkier nature. At a higher discharge product growth (particularly at O_2_ = 500, blue curve in Figure 8a–d), DMSO is pushed out of the micropores into the slit-mesopore region (i.e., the center of the graph) due to the formation of large-sized clusters. This supports the hypothesis that once the solubility limit of the discharge product (Li_2_O_2_) in the electrolyte is reached (i.e., 0.09 mol·m^–3^), the precipitation of the solid product takes place, leading to its deposition inside the pores.^64^ It was also noticed that among the studied frameworks, RHO-ZTC is the only structure capable of maintaining DMSO in micropores at a high oxygen content (Figure 8a).

### 3.4 Discharge Product Cluster Formation

**Figure 9:**
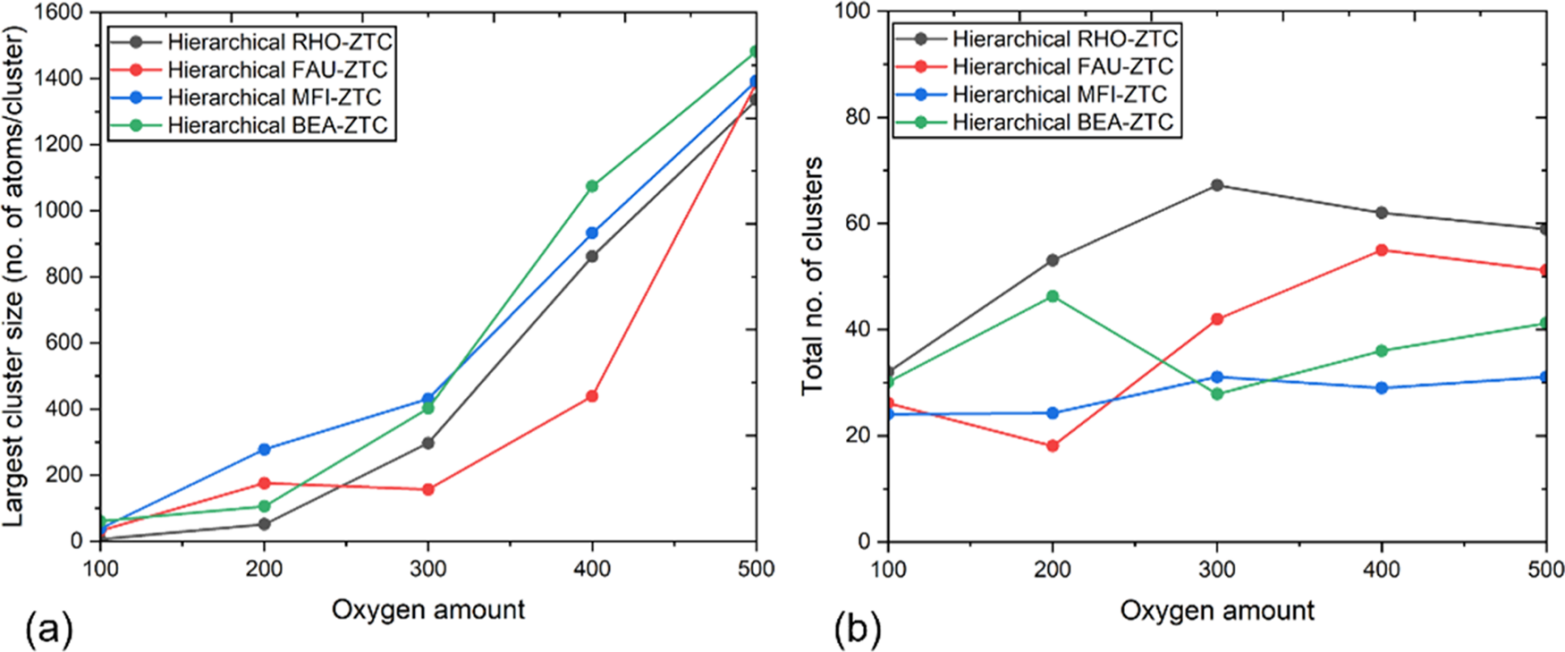
(a) Evolution of largest cluster size and (b) total number of clusters formed through the discharging of hierarchical ZTC-based electrodes.

To gain qualitative insights into the cluster formation of the discharge product (Li_2_O_2_), we performed cluster analysis based on bond connectivity. Figure 9a illustrates the size evolution of the largest cluster formed in the hierarchical ZTC electrodes. It depicts a gradual increase in the aggregate size of the number of oxygen molecules. Throughout the discharge process, hierarchical RHO-ZTC (black curve) shows the largest number (Figure 9b) of smaller clusters compared to the other studied morphologies. This is due to the presence of a large pore size in RHO-ZTC as well as an easy access of liquid electrolyte through micropores, which may wash away part of the discharge products. Consequently, RHO-ZTC promotes a higher diffusivity of oxygen and lithium inside RHO-ZTC than the FAU, MFI, and BEA structures, which forms large aggregates of discharge products. At high oxygen consumption (i.e., O_2_ = 500 molecules), hierarchical BEA-ZTC has a larger cluster size (see Figure 9a) than the rest of the structures. This is due to fewer electrolyte density numbers present inside small-sized microporous walls, leading to the continuous growth of discharge product aggregates that are difficult to break by a limited amount of aprotic electrolyte (i.e., DMSO). Besides, Figure 9b illustrates that the hierarchical ZTCs having micropores >9 Å (particularly RHO-ZTC and FAU-ZTC) inhibit the generation of large aggregates due to the presence of an excess amount of electrolyte solution inside the micropores. However, this is not the case for MFI-ZTC and BEA-ZTC frameworks (with micropores <9 Å) in which electrolyte transport into microporous walls is more difficult, resulting in reducing the total number of clusters to synthesize large clusters. From the preceding discussion, we have deduced that pores mainly influence the size and number of Li_2_O_2_ product clusters. For instance, larger pores (specifically in hierarchical RHO-ZTC) lead to the formation of smaller-sized clusters, preventing pore clogging and facilitating prolonged mass transport within the structure’s pores. Consequently, these larger pores provide ample space for storing the discharge Li_2_O_2_, resulting in a higher discharge capacity. On the contrary, smaller pores tend to produce larger discharge product (Li_2_O_2_) clusters, mainly due to the strong interactions between the product molecules and pore walls. Therefore, they are more prone to early choking due to the larger clusters, resulting in limited mass transport and a lower discharge capacity.

**Figure 10:**
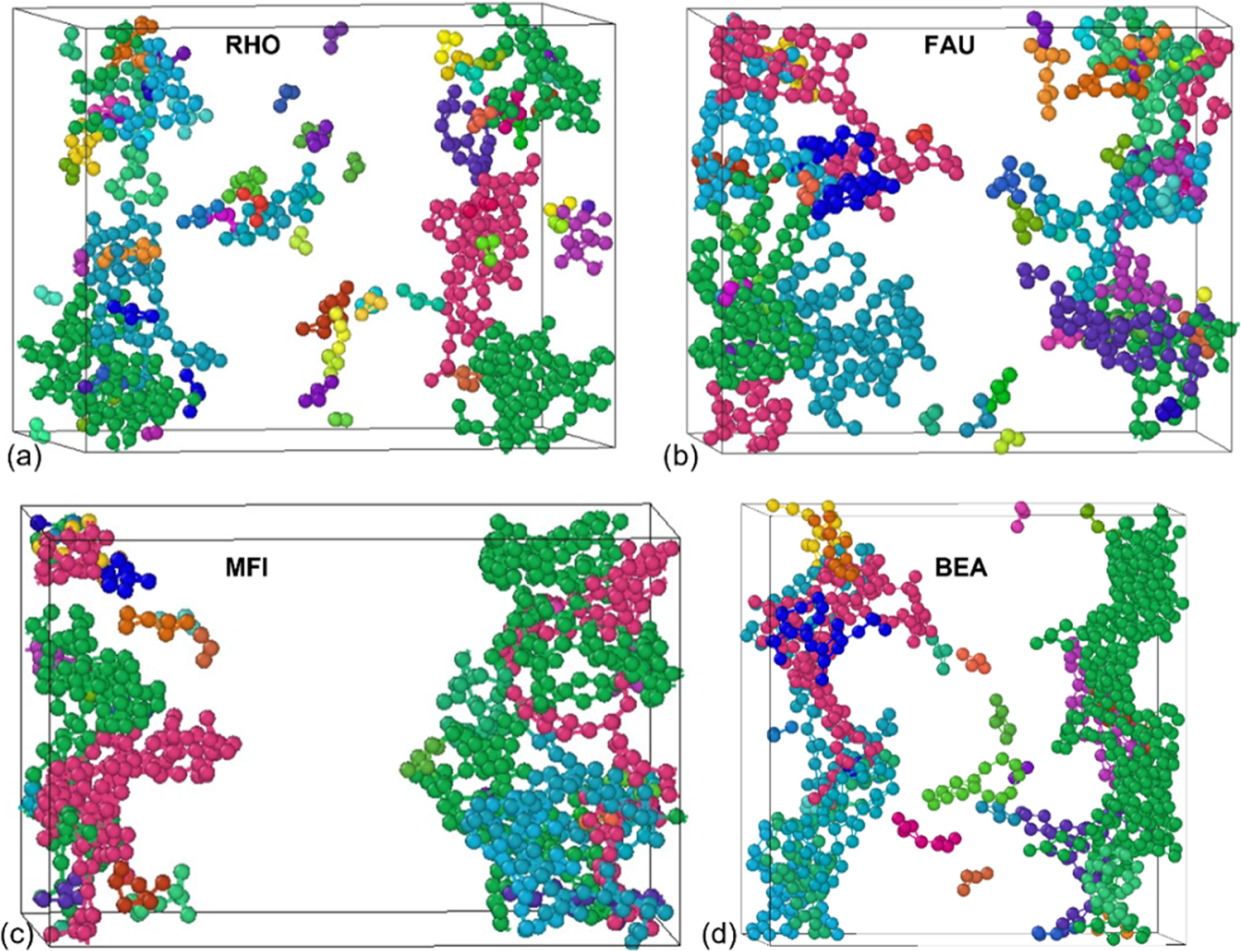
Visuals of discharge product clusters forming in hierarchical ZTCs: (a) RHO, (b) FAU, (c) MFI, and (d) BEA. Dark green represents the largest cluster, and yellow represents the smallest cluster.

As an example, Figure 10 shows the visuals of the discharge product cluster formation at oxygen consumption of 300 molecules through all four hierarchical ZTCs. For the other cases (i.e., 100, 200, and 500 oxygen molecules in the system), visuals of large aggregates can be found in the Supporting Information (Figure S4). Figure 10a–d reveals that clusters of different sizes are formed with the smallest clusters consisting of single Li_2_O_2_, which represent the basic discharge product generated in the hierarchical cathodes. We have observed that the formation of large product aggregates occurs through the addition of either LiO_2_^–^ and Li^+^ separately or in conjunction with the individual Li_2_O_2_ product. Initially, lithium superoxide (LiO_2_^–^) is formed from the rapid reaction of Li^+^ and O_2_^2–^. This is followed by a slower reaction (with Li^+^) that generates lithium peroxide (Li_2_O_2_),^65^ as explained in Table S6.

Furthermore, initially, a greater formation of lithium superoxide (LiO_2_^–^) molecules takes pace leading to lithium peroxide (Li_2_O_2_) product clusters via solution-mediated growth rather than surface deposition. This is due to the system having excess Li^+^, which preferably reacts with LiO_2_^–^ (present in the electrolyte solution) forming the Li_2_O_2_ product. This finding agrees with the density functional theory study performed by Lu et al.,^65^ who claimed that oxygen and lithium cations promote the formation of solvated LiO_2_ in the presence of a single electron. This can be further reduced via two mechanisms: (1) cluster formation once the electrolyte solution becomes supersaturated with LiO_2_ molecules or (2) disproportionation of the LiO_2_ dimer [(LiO_2_)_2_] to Li_2_O_2_ and O_2_. In this work, we have mimicked both reactions with the dominancy of the former mechanism because of the presence of excess numbers of Li^+^, which quickly react with the LiO_2_^–^ individual molecules, thereby giving the subsequent clusters of Li_2_O_2_ (Table S6).

## 4 Conclusions

Novel hierarchical zeolite-templated carbon electrodes were screened and analyzed to investigate their potential as an electrode architecture for Li–O_2_ batteries with an enhanced discharge capacity. The structural properties of the materials were characterized by nitrogen adsorption using GCMC simulations. The performance of the promising four distinct ZTC structures, namely, RHO-, BEA-, MFI-, and FAU-ZTCs was investigated by using reaxFF-MD. The transport properties of 1 M LiPF_6_ electrolyte (solvent: DMSO) solution inside hierarchical zeolite-templated carbon electrodes were simulated by changing the oxygen concentration. The results indicate that the RHO-ZTC electrode enhanced mass transfer compared to conventional microporous ZTC (approximately 31% for O_2_, 44% for Li^+^, and 91% for DMSO) electrodes. It resulted in lithium ion and oxygen self-diffusivities of 1.75 × 10^–10^–2.69 × 10^–11^ m^2^·s^–1^ and 1.17 × 10^–10^–1.53 × 10^–11^ m^2^·s^–1^, respectively. This is due to the increase in the number of reaction sites inside the microporous region while keeping sufficient free space for oxygen transport in the porous channel.

The density number profiles of Li^+^ and O_2_ depicted that these particles preferentially dwell within the microporous walls, indicating the oxygen reduction reaction (ORR) and deposition of discharge product within the micropores, while the slit-type mesoporous region acts as an oxygen transport tunnel to continue oxygen supply into the micropores for the efficient utilization of hierarchical electrodes. This claim is also supported by the outflow of electrolyte (at a high discharge rate) to the mesopore from microporous walls. Further, the cluster analysis revealed that the presence of electrolyte inside micropores >9 Å (particularly, hierarchical RHO-ZTC and FAU-ZTC structures) inhibit the formation of large-sized aggregates of discharge products. It was found that discharge product clusters are composed of Li_2_O_2_. This finding strengthens the hypothesis that hierarchical air electrodes with a tailored framework might guide to enhance the discharge performance of the Li–O_2_ battery.

Future Li–O_2_ battery research can focus on developing an integrated method to examine the impact of both micro- and mesopores on the cell-level performance. Additionally, the impact of air impurities (H_2_O, CO_2_, and N_2_) on the oxygen reduction reaction, discharge product clustering, and overall performance of Li–air batteries should be investigated in detail, as a matter of future work.

## Supporting Information Available

Additional structural property data of the selected 47 ZTCs; illustrations demonstrating the construction of ZTC materials; input data for cohesive energy in MD simulations; detailed simulation results for the self-diffusivities of various species; visual representation of the formation of discharge product clusters; methods employed for mimicking reactions in MD simulations (PDF)
