# Supplementary material for: Exploring the Potential of Hierarchical Zeolite-Templated Carbon Materials for High-Performance Li–O2 Batteries: Insights from Molecular Simulations
Source: ACS Appl Mater Interfaces. 2023 Nov 16;15(47):54432–45. doi: 10.1021/acsami.3c11586 (PMC10694818; doi:10.1021/acsami.3c11586)
Supplement: Supplementary file 1 — am3c11586_si_001.pdf [file am3c11586_si_001.pdf]

Supporting Information

Exploring the potential of Hierarchical Zeolite-templated Carbon materials for high performance Li-O_2 Batteries: Insights from molecular simulations

Khizar Hayat, Daniel Bahamon*, Lourdes F. Vega, Ahmed AlHajaj*

Research and Innovation Center on CO_2 and Hydrogen (RICH Center) and Chemical Engineering Department, Khalifa University, P.O. Box 127788, United Arab Emirates.

* Corresponding Author: daniel.garcia@ku.ac.ae and ahmed.alhajaj@ku.ac.ae

Table of contents

S 1. Structural properties data of selected 47 ZTC S-2

S 2. Illustrations demonstrating the construction of ZTC materials S-4

S 3. Input data for Cohesive Energy in MD simulations S-5

S 4. Simulation results for O_2, Li, and DMSO diffusivities S-5

S 5. Visual representation of the formation of discharge product clusters S-8

S 6. Methods employed for mimicking reactions in MD simulations S-9

S 1. Structural properties data of selected 47 ZTC

Table S1. Structural analysis results of microporous ZTCs database

ZTC	A (m^2g^{-1})	V_p (cm^3g^{-1})	PLD (Å)	LCD (Å)
AEI	451.48	0.319	3.57	5.59
BEA	1276.19	0.414	5.85	7.49
BEC	1546.96	0.512	6.1	7.53
IFU	1282.8	0.491	6.85	9.69
ISV	1614.92	0.55	6.43	8.57
ITG	1464	0.513	6.25	7.9
ITH	1767.58	0.6	6.53	8.36
ITR	1742	0.637	5.72	8.29
IWR	1637.37	0.518	5.81	7.86
IWS	1371.15	0.476	6.45	11.26
IWW	2248.31	0.778	6.9	9.91
JSR	185.22	0.155	2.8	6.75
MEL	2853.68	0.885	6.59	8.38
MFI	2552.37	0.731	5.9	7.46
MSE	1555.21	0.56	5.7	10.06
POS	1384.06	0.473	6.52	7.86
PUN	21.76	0.122	2.84	4.64
RHO	2499.21	1.139	9.8	14.95
RWY	590.9	0.36	4.07	7.68
SAO	1583.19	0.516	6.78	8.63
SZR	0	0	2.11	3.19
TUN	2653.78	0.809	6.8	9.19
UOV	1876.12	0.643	7.01	9.77
UWY	1820.64	0.624	7.23	9.51
AFY	503.08	0.25	4.24	5.73
EMT	1776.56	0.7	8.66	10.82
EWT	1434.62	0.496	6.19	8.89
FAU	1664.32	0.697	9.08	11.01
BEB	1562.37	0.507	5.71	7.83
BOG	206.29	0.185	4.39	5.42
BSV	227.27	0.208	2.97	5.31
DFO	703.15	0.332	4.69	6.74
ERI	967.04	0.394	5.64	7.05
GME	74.93	0.197	2.53	4.77
IRR	1280.43	0.528	6.92	10.2
IRY	1098.6	0.439	7.1	9.47
ITN	1514.28	0.504	6.39	8.4
ITT	1029.84	0.425	4.93	9.3
LTA	1967.51	0.653	5.24	7.41
OFF	850.97	0.332	6.61	6.94
OSO	0	0	2.08	2.94
SBS	1741.72	0.615	6.75	10.03
SBT	1659.76	0.588	6.19	9.9

SOF	1741.43	0.614	5.81	8.42
STW	183.25	0.323	3.53	5.15
SVR	3300.83	0.957	6.63	8.08

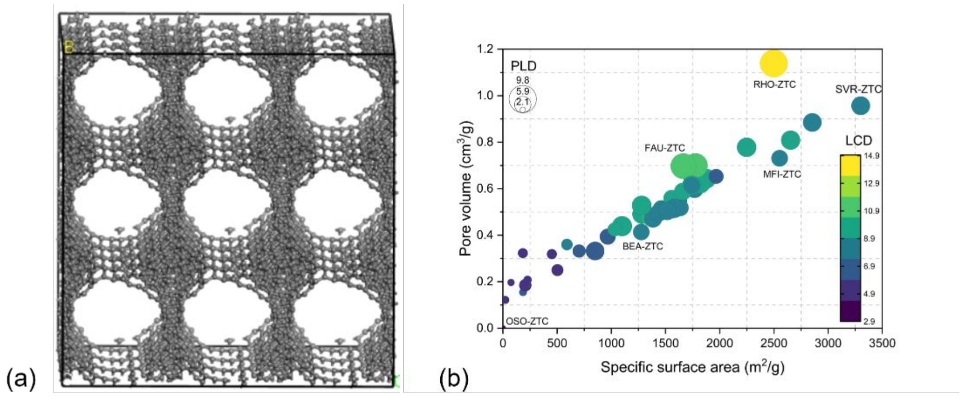

Figure S1. (a-b) Structural properties of selected 47 ZTCs.

S 2. Illustrations demonstrating the construction of ZTC materials

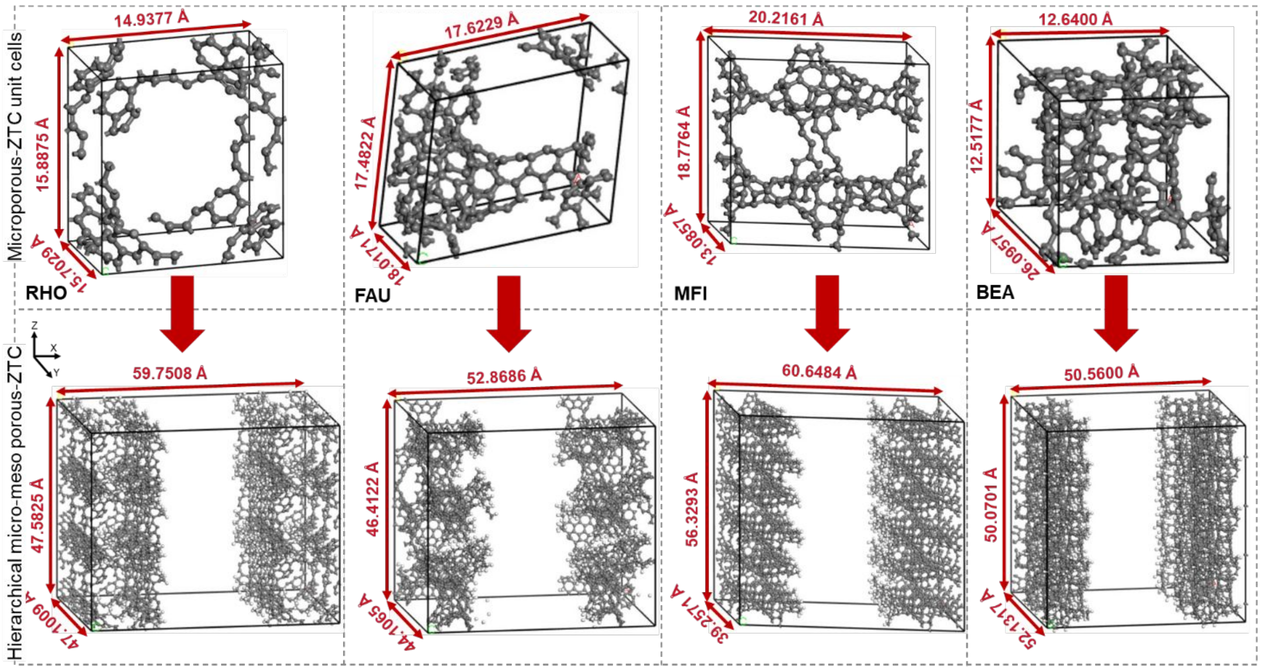

Figure S2. Generation procedure of hierarchical ZTCs from their microporous parent structures.

S 3. Input data for Cohesive Energy in MD simulations

Table S2. Cohesive energies of the hierarchical ZTCs and their microporous parent structures

Structure	Parent ZTC unit cell				Hierarchical ZTC supercell			
	n	m	$n+m$	E_{coh} (eV/atom)	n	m	$n+m$	E_{coh} (eV/atom)
BEA	240	-	240	-8.293	3976	1032	5008	-6.579
FAU	176	-	176	-7.825	2625	664	3289	-6.643
MFI	210	-	210	-8.026	3360	440	3800	-7.080
RHO	118	-	118	-8.082	2781	1080	3861	-5.869

E_C = -0.027 (eV) and E_H = -2.357 (eV)

S 4. Simulation results for O_2, Li, and DMSO diffusivities

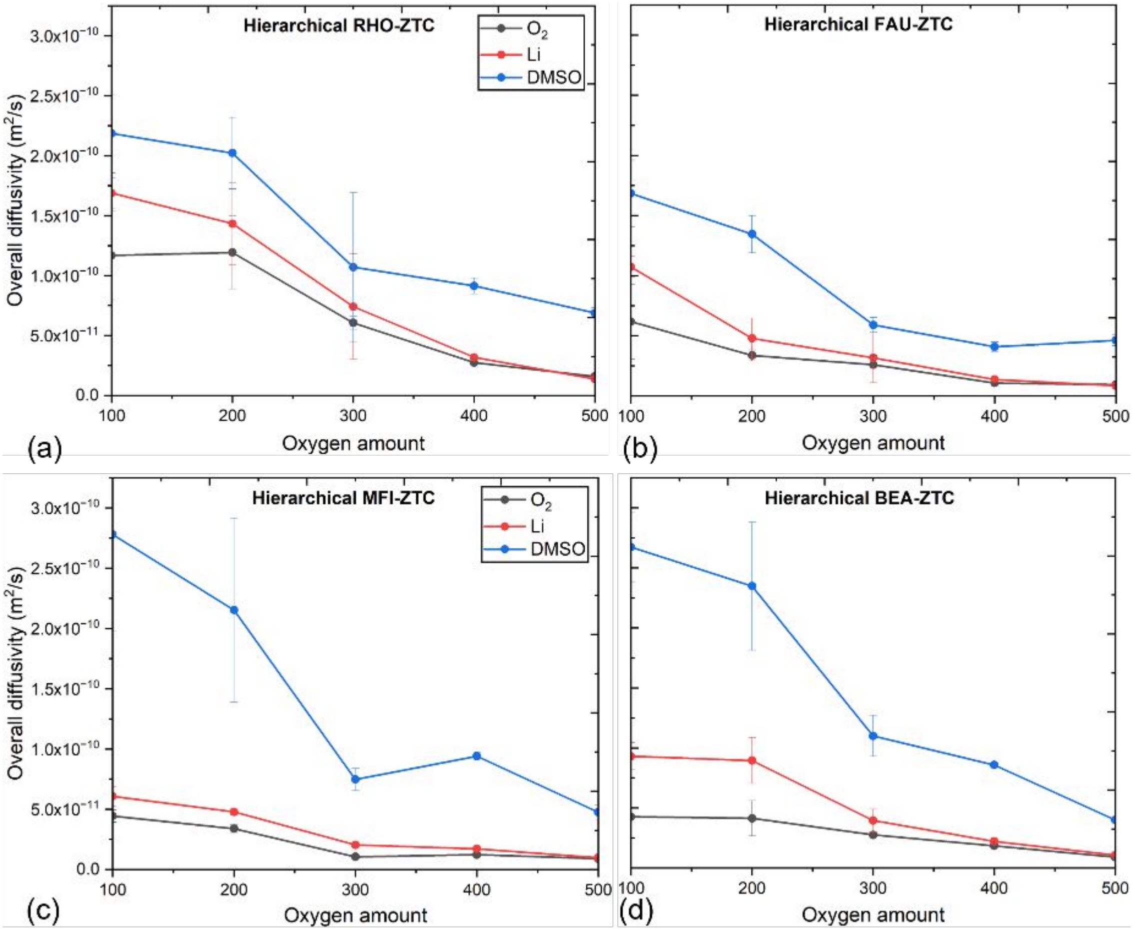

Figure S3. Simulation results of overall self-diffusivities of species through hierarchical (a) RHO-ZTC, (b) FAU-ZTC, (c) MFI-ZTC, and (d) BEA-ZTC

Table S3. Self-diffusivity results (D_x ×10^{10} m^2/s) of O_2, Li, and DMSO through hierarchical ZTCs along x-direction

	Hierarchical RHO-ZTC					Hierarchical FAU-ZTC					Ref.
O_2	100	200	300	400	500	100	200	300	400	500	
D_{O2}	1.170 ± 0.27	0.997 ± 0.12	0.680 ± 0.14	0.265 ± 0.03	0.153 ± 0.01	5.590 ± 0.17	0.337 ± 0.01	0.259 ± 0.02	0.070 ± 0.07	0.094 ± 0.002	-
D_{Li}	1.750 ± 0.11	1.340 ± 0.21	0.768 ± 0.07	0.315 ± 0.03	0.269 ± 0.003	0.693 ± 0.09	0.479 ± 0.03	0.317 ± 0.05	0.095 ± 0.01	0.084 ± 0.02	0.306 ± 0.10 [25]
D_{DMSO}	2.440b ± 0.16	2.050 ± 0.14	1.070 ± 0.07	0.959 ± 0.03	0.655 ± 0.07	1.810 ± 0.18	1.350 ± 0.16	0.544 ± 0.25	0.394 ± 0.01	0.441 ± 0.06	1.465 ± 0.10 [25]
	Hierarchical MFI-ZTC					Hierarchical BEA-ZTC					
O_2	100	200	300	400	500	100	200	300	400	500	
D_{O2}	4.300 ± 0.10	0.268 ± 0.007	0.133 ± 0.008	0.106 ± 0.02	0.090 ± 0.007	0.559 ± 0.23	0.414 ± 0.05	0.275 ± 0.01	0.152 ± 0.01	0.090 ± 0.001	
D_{Li}	0.608 ± 0.17	0.477 ± 0.05	0.275 ± 0.03	0.178 ± 0.03	0.098 ± 0.002	1.090 ± 0.13	0.872 ± 0.12	0.536 ± 0.01	0.192 ± 0.02	0.103 ± 0.002	
D_{DMSO}	2.840 ± 0.02	1.550 ± 0.15	0.849 ± 0.11	0.769 ± 0.13	0.418 ± 0.02	2.130 ± 0.19	1.840 ± 0.21	0.949 ± 0.08	0.807 ± 0.14	0.377 ± 0.02	

Table S4. Overall self-diffusivities (D ×10^{10} m^2/s) of O_2, Li, and DMSO through hierarchical ZTCs

	Hierarchical RHO-ZTC					Hierarchical FAU-ZTC					Ref.
O_2	100	200	300	400	500	100	200	300	400	500	
D_{O2}	1.170 ± 0.31	1.190 ± 0.25	0.606 ± 0.08	0.274 ± 0.09	0.159 ± 0.11	0.617 ± 0.50	0.338 ± 0.13	0.259 ± 0.30	0.108 ± 0.02	0.094 ± 0.02	
D_{Li}	1.680 ± 0.07	1.430 ± 0.23	0.741 ± 0.59	0.318 ± 0.02	0.137 ± 0.05	1.070 ± 0.08	0.479 ± 0.34	0.318 ± 0.64	0.137 ± 0.009	0.084 ± 0.25	2.44 ± 0.05 [25]<br>4.4 ± 0.10 [49]
D_{DMSO}	2.180 ± 0.15	2.020 ± 0.14	1.070 ± 0.57	0.914 ± 0.07	0.688 ± 0.06	1.680 ± 0.16	1.350 ± 0.11	0.591 ± 0.10	0.408 ± 0.09	0.463 ± 0.09	8.25 ± 0.010 [25]

	Hierarchical MFI-ZTC					Hierarchical BEA-ZTC				
O_2	100	200	300	400	500	100	200	300	400	500
D_{O2}	0.444 ± 0.11	0.339 ± 0.09	0.106 ± 0.01	0.123 ± 0.01	0.090 ± 0.03	0.426 ± 0.09	0.414 ± 0.35	0.276 ± 0.06	0.185 ± 0.04	0.090 ± 0.01
D_{Li}	0.607 ± 0.13	0.478 ± 0.05	0.205 ± 0.01	0.172 ± 0.008	0.098 ± 0.08	0.931 ± 0.12	0.895 ± 0.21	0.395 ± 0.23	0.222 ± 0.13	0.107 ± 0.02
D_{DMSO}	2.780 ± 0.28	2.150 ± 0.35	0.747 ± 0.12	0.941 ± 0.03	0.475 ± 0.12	2.670 ± 0.10	2.350 ± 0.22	1.100 ± 0.15	0.858 ± 0.02	0.400 ± 0.05

Table S5. Comparative analysis of self-diffusivities ($D_x \times 10^{10}$ m^2/s) through hierarchical and microporous RHO- ZTCs along x-direction

	Hierarchical RHO-ZTC				Parent RHO-ZTC			
O_2 concentration	2461	4923	7385	12309	2461	4923	7385	12309
D_{O2}	1.170 ± 0.27	0.997 ± 0.12	0.680 ± 0.14	0.153 ± 0.01	0.680 ± 0.21	0.905 ± 0.33	0.615 ± 0.08	0.383 ± 0.05
D_{Li}	1.750 ± 0.11	1.340 ± 0.21	0.768 ± 0.07	0.269 ± 0.003	0.975 ± 0.06	1.140 ± 0.10	0.875 ± 0.02	0.539 ± 0.19
D_{DMSO}	2.440b± 0.16	2.050 ± 0.14	1.070 ± 0.07	0.655 ± 0.07	0.198 ± 0.18	1.560 ± 0.16	0.697 ± 0.03	0.660 ± 0.05

S 5. Visual representation of the formation of discharge product clusters

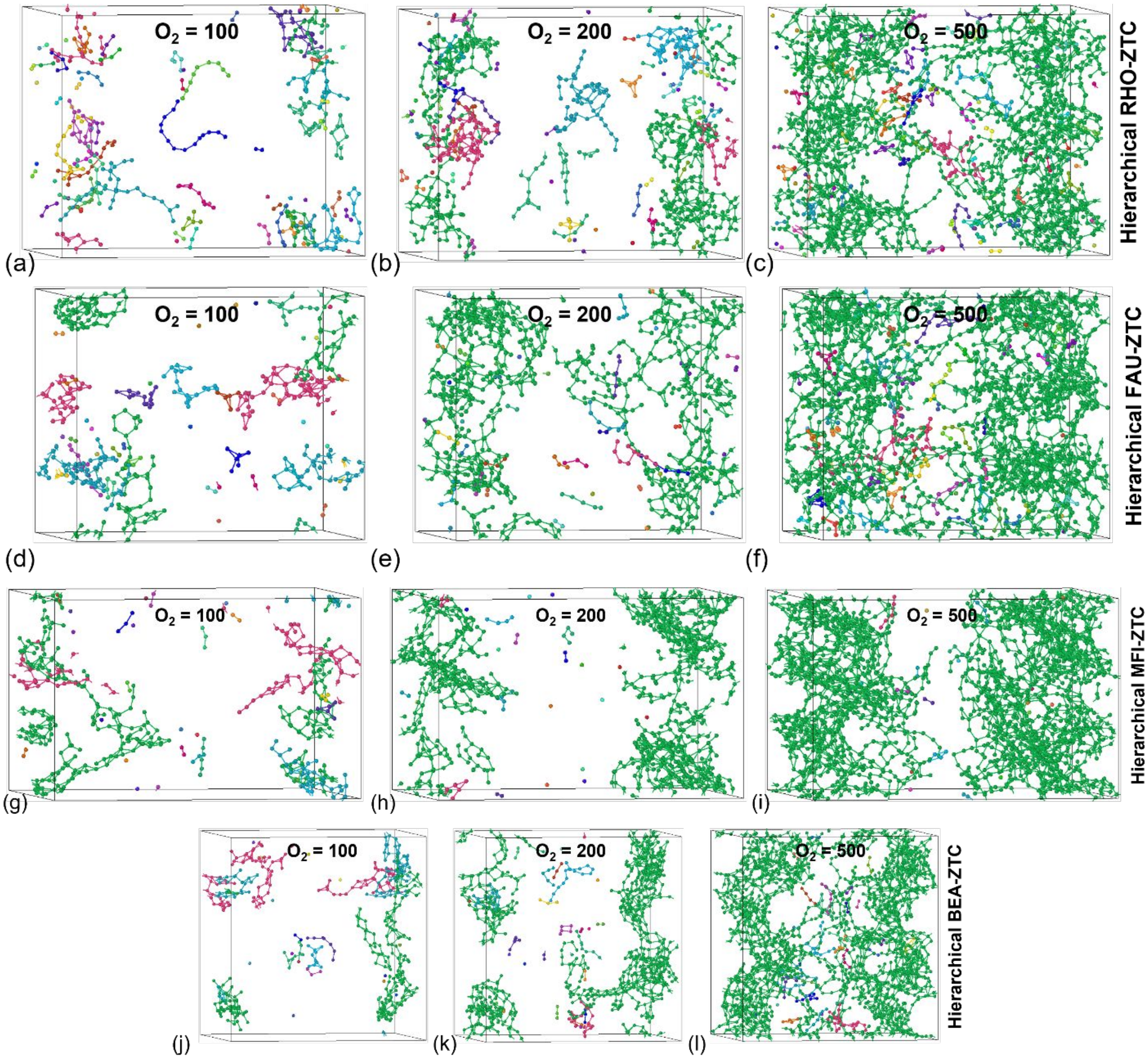

Figure S4. Discharge product clusters formation at various oxygen consumptions (100, 200, and 500) through hierarchical (a-c) RHO-ZTC, (d-f) FAU-ZTC, (g-i) MFI-ZTC, and (j-l) BEA-ZTC. Snapshots after production runs (1.5 ns). Other molecules (electrolyte and PF6) are omitted

S 6. Methods employed for mimicking reactions in MD simulations

Table S6. Mimicking of experimental reactions in reaxFF-MD simulations

Reaction mechanism in experiments	Mimicked mechanism in reaxFF-MD
$O_2+e^- \rightarrow O_2^-$	
$Li^++O_2^- \rightarrow LiO_2$	$Li^++O_2^{2-} \rightarrow LiO_2^-$
$LiO_2+Li^+ +e^- \rightarrow Li_2O_2$	$LiO_2^-+Li^+ \rightarrow Li_2O_2$
**OR** $2LiO_2 \rightarrow Li_2O_2+O_2$	**OR** $2LiO_2^- \rightarrow Li_2O_2+O_2^{2-}$
